# Population based germline testing for primary cancer prevention

**DOI:** 10.18632/oncotarget.25995

**Published:** 2018-09-04

**Authors:** Ranjit Manchanda, Rosa Legood

**Affiliations:** Ranjit Manchanda: Barts Cancer Institute, Queen Mary University of London, Charterhouse Square, London, UK; Department of Gynaecological Oncology, St Bartholomew’s Hospital, London, UK; Gynaecological Cancer Research Centre, Department of Women’s Cancer, Institute for Women’s Health, University College London, London, UK; Rosa Legood: Department of Health Services Research and Policy, London School of Hygiene & Tropical Medicine, London, UK

**Keywords:** population based testing, BRCA, breast cancer, ovarian cancer, genetic testing

Next generation sequencing technologies [1, 2], increasing affordability, the ability to undertake high throughput large volume testing and advances in bioinformatics has made large scale population based genetic testing technically feasible. Coupled with a rapidly changing genomic landscape, improved genetic understanding of disease and increasing awareness, this offers a massive opportunity to apply this knowledge and technology on a broad population scale to make an important shift in healthcare towards disease prevention. Primary prevention and early detection strategies remain the cornerstone for reducing the burden of cancers in the population and this underpins the clinical utility of genetic testing for moderate/high penetrance cancer gene mutations.

The traditional approach to genetic-testing is based on an *a priori* threshold of mutation prevalence which is calculated using clinical-criteria centred mainly on family-history (FH). For *BRCA*-mutations this used to be set at 20% probability, which was subsequently reduced to around 10% probability. Approaches in use in clinical practice for this purpose range from assessing the FH through number of standardised clinical criteria to complex prediction models. This clinical-criteria/FH-based approach has a number of limitations. It needs family members to be aware of the FH of cancer, and both members of the family and the GP/health professional consulted to understand the importance of this history and then make the referral to a genetics centre. We and others have shown this gate keeper approach misses over half the mutation carriers at risk. Small families, poor communication, paternal inheritance, limited awareness, etc. contribute to the poor performance of criteria based testing in ruling out the presence of a mutation. The current system is associated with huge underutilisation of genetic testing and delayed identification of unaffected individuals [3, 4]. Over 80% of eligible patients using National Cancer Comprehensive Network criteria in the USA have not been referred/undergone testing. We recently estimated that only 3% of the estimated *BRCA*-carriers across Greater London (16-million population) had been identified and using forecasting models showed the current rates of testing are inadequate to detect all *BRCA*-carriers in the population [4]. A number of these limitations can be overcome by removing restrictions, broadening access and offering testing to everyone, i.e. population-testing.

Population based *BRCA*-testing in the Jewish population has been thoroughly investigated in the UK Genetic Cancer Prediction through Population Screening (GCaPPS) randomised-controlled trial (RCT) (ISRCTN73338115) [5], as well as in Israeli [6] and Canadian [7] single-arm cohort studies. Data show that in the Jewish-population unselected *BRCA*-testing identifies >50% additional BRCA-carriers, is acceptable, feasible, can be undertaken in a community setting and does not adversely affect psychological well-being or quality-of-life compared to FH-based testing, and has high satisfaction rates [5, 8]. Additionally such an approach is highly cost-effective for both UK and US health systems [9-11]. In fact it is cost-saving in most scenarios. There is thus strong evidence to support change in paradigm to population-based *BRCA*-testing in the Jewish community. However, these data cannot be directly extrapolated to the non-Jewish general population.

Nevertheless, general (non-Jewish) population testing offers the opportunity to use genomics to maximise cancer prevention/early detection and reduce cancer burden on a much larger scale. Why should individuals in the family need to develop cancer before other unaffected individuals in the family can be identified? Additionally, the availability of panel germline testing now enables population testing to be undertaken for multiple cancer susceptibility genes but the genes included must have ‘well-established’ clinical-utility. Surgical prevention is cost-effective [12, 13] for the newer moderate penetrance OC gene mutations RAD51C/RAD51D/BRIP1 (OC lifetime risks ∼6-11%) and testing for these is now established in clinical practice [14]. PALB2 is a moderate-risk gene with BC-risks [15] for which MRI/mammogram screening and risk-reducing mastectomy is available. In addition the Lynch Syndrome associated genes MLH1, MSH2 and MSH6 are also potential candidates that could become part of an extended population germline testing panel.

We recently modelled population testing for multiple BC and OC gene mutations (BRCA1/BRCA2/RAD51C/RAD51D/BRIP1/PALB2) compared to the traditional clinical-criteria/FH-based approach [16]. We showed that panel testing for multiple OC/BC genes was more cost-effective than *BRCA*-testing alone. Critically, population based testing for BRCA1/BRCA2/RAD51C/RAD51D/BRIP1/PALB2 was more cost-effective than any currently used clinical-criteria/family-history based strategy. The ICER (incremental cost-effectiveness ratio) was £21,599.96/QALY and $54,769.78/QALY for UK and US health systems, well below the thresholds of £30,000/QALY and $100,000/QALY in the UK and USA respectively. This amounts to 9.34 or 7.57 days life-expectancy gained (across the population). Robust sensitivity analyses (10,000 simulations on probabilistic sensitivity analysis) indicated that population testing is cost-effective and the preferred strategy in 84% simulations in the UK and 93% simulations in the USA models respectively. Such an approach could potentially prevent an additional 657/655 OC-cases and 2420/2386 BC cases per million UK/USA women respectively. Extrapolating this across the population amounts to potentially 17505 and 65221 OC cases prevented in UK and USA women and 64493 and 237610 BC cases prevented in UK and USA women respectively. However, cost-effectiveness analysis, incurs assumptions, and further research is necessary to prospectively validate some key assumptions, such as, the surgical prevention uptake rates in those without a strong FH of cancer.

The feasibility of population based germline panel testing for OC gene mutations has been demonstrated in an ongoing pilot study in London [17]. The time has come to undertake large research studies to evaluate the impact of population based panel germline testing in an unselected non-Jewish general population. This includes impact on quality-of-life, psychological well-being, satisfaction and long-term health-behaviour and lifestyle. The best implementation model to deliver this approach also needs to be identified through robust evaluation and comparison with the current standard of care in well-designed trial(s). A key issue which will need resolving is a system for ongoing monitoring, reclassification (where needed) and management of VUS (variants of uncertain significance). Other matters that need tackling include increasing public/health professional awareness and education, delivery logistics, quality-control, call-recall mechanisms and expansion of downstream pathways of care. These steps are necessary for the health-system to achieve its maximum potential for reducing burden of disease now afforded through cancer screening and prevention.
